# Smoking and Physical Activity Trajectories from Childhood to Midlife

**DOI:** 10.3390/ijerph16060974

**Published:** 2019-03-18

**Authors:** Kasper Salin, Anna Kankaanpää, Mirja Hirvensalo, Irinja Lounassalo, Xiaolin Yang, Costan G. Magnussen, Nina Hutri-Kähönen, Suvi Rovio, Jorma Viikari, Olli T. Raitakari, Tuija H. Tammelin

**Affiliations:** 1 Faculty of Sport and Health Sciences, University of Jyväskylä, 40014 Jyväskylä, Finland; Mirja.hirvensalo@jyu.fi (M.H.); irinja.lounassalo@jyu.fi (I.L.); 2 LIKES Research Centre for Physical Activity and Health, 40014 Jyväskylä, Finland; anna.kankaanpaa@likes.fi (A.K.); xiaolin.yang@likes.fi (X.Y.); tuija.tammelin@likes.fi (T.H.T.); 3 Research Centre of Applied and Preventive Cardiovascular Medicine, University of Turku, 20520 Turku, Finland; cmagnuss@utas.edu.au (C.G.M.); suvi.rovio@utu.fi (S.R.); olli.raitakari@utu.fi (O.T.R.); 4 Menzies Institute for Medical Research, University of Tasmania, 7005 Hobart, Australia; 5 Department of Pediatrics, University of Tampere and Tampere University Hospital, 33100 Tampere, Finland; nina.hutri-kahonen@uta.fi; 6 Department of Medicine and Division of Medicine, University of Turku and Turku University Hospital, 20500 Turku, Finland; jorma.viikari@utu.fi; 7 Department of Clinical Physiology and Nuclear Medicine, Turku University Hospital, 20500 Turku, Finland

**Keywords:** physical activity, smoking, cohort study, longitudinal study, trajectory, adults

## Abstract

*Introduction:* Despite substantial interest in the development of health behaviors, there is limited research that has examined the longitudinal relationship between physical activity (PA) and smoking trajectories from youth to adulthood in a Finnish population. This study aimed to identify trajectories of smoking and PA for males and females, and study the relationship between these trajectories from youth to adulthood. *Methods:* Latent profile analysis (LPA) was used to identify trajectories of smoking and PA separately for males and females among 3355 Finnish adults (52.1% females). Participants’ smoking and PA were assessed five to eight times over a 31-year period (3–18 years old at the baseline, 34–49 years at last follow-up). Multinomial logistic regression analysis was used to study the relationship between the trajectories of smoking and PA. *Results:* Five smoking trajectories and four to five PA trajectories were identified for males and females. Of the PA trajectory groups, the persistently active group were least likely to follow the trajectories of regular smoking and the inactive and low active groups were least likely to follow non-smoking trajectory group. Likewise, inactive (women only) and low active groups were less likely to belong to the non-smokers group. *Conclusions:* The study suggests that those who are persistently active or increasingly active have substantially reduced probabilities of being in the highest-risk smoking categories.

## 1. Introduction

Smoking has been, and remains, a major risk factor for human health [1]. In addition, there are many countries, including populous ones like Indonesia and Pakistan, where tobacco smoking is projected to continue increasing at least until 2025, as well as countries where there has been an explosion of smoking, such as Cameroon or Congo [2]. To promote the reduction and cessation of smoking, it is essential to understand smoking behavior and other health behaviors that may be a gateway for a non-smoking lifestyle. Lack of physical activity (PA) is a risk factor for several major health problems. These include more than 25 chronic conditions such as cardiovascular disease, diabetes mellitus, depression, and several cancers [3]. Because smoking is also a major risk factor for several of these diseases [4], physically inactive smokers are at an even higher risk of developing these diseases [5]. Smoking has also been seen as a predictor of other unhealthy behaviors [6], and PA has been suggested to be a “gateway” to healthier habits, meaning that when changes in PA occur, other changes will follow [7]. On the other hand, smoking and PA have been viewed largely as independent behaviors, with changes in one behavior not necessarily tied to a change in the other [8].

PA in adolescence tracks into adulthood [9], with low PA and inactivity tending to track better than high PA [10]. Similarly, smoking in adolescence tends to continue in young adulthood and it has been shown to have the strongest level of continuity into adulthood when compared to other health habits such as PA and alcohol use [11]. Additionally, previous studies have also found associations between unhealthy habits. For example, smoking in adolescence is associated with alcohol use, physical inactivity, and poor dietary behavior in adulthood [6,12], as well as illicit drug use, unprotected sexual intercourse, and aggressive behavior [13]. Risky health behaviors have also been found to be associated with gender and sociodemographics, with males showing riskier health behaviors than women [14,15].

To analyze how individual characteristics or behaviors are organized into meaningful patterns that distinguish subgroups of people, mixture modeling is a useful technique [16,17,18]. This technique allows identifying unobserved subgroups in the population following distinct developmental trajectories or patterns [18].

Previous studies on smoking show evidence for two [19], three [20], four [21,22], five [23,24], and six [25] distinct smoking trajectory groups. In these studies, three common smoking trajectories were found: (1) never or rare smokers, (2) persistent light smokers, and (3) stable or steady heavy smokers. Other trajectories that have been identified are early/late increasers, decreasers [22,24,25], and quitters [23].

Likewise, in previous longitudinal studies concerning PA, participants have been grouped into three [26], four [27], or five [28,29] trajectory groups. In these studies, three main PA trajectory groups were identified: inactive, moderately active, and highly active [26]. Other trajectories that have been identified include increasing, decreasing, or curvilinear PA groups [27,28].

Although cross-sectional studies have examined the clustering of health habits, there is a gap in the research literature identifying the linear change of the longitudinal research related to the association between smoking and PA. Even though there are some previous studies, with comprehensive data, about the association of PA and smoking, these rely mainly on longitudinal data collected from youth [28,30,31,32,33], but not from youth to adulthood. This study fills the gap in the research literature from youth to adulthood. Using data from the 31-year prospective Cardiovascular Risk in Young Finns Study, we aimed to (1) identify different subgroups of participants who shared similar smoking and PA trajectories from youth to mid-adulthood, and (2) determine the associations between the identified smoking and PA trajectories from youth to adulthood.

## 2. Methods

### 2.1. Study Design and Participants

The data were obtained from the Cardiovascular Risk in Young Finns Study. The population at baseline (1980) consisted of boys and girls aged 3, 6, 9, 12, 15 and 18 years and randomly sampled from all five Finnish university cities with medical schools (Helsinki, Turku, Tampere, Oulu, and Kuopio) and their rural surroundings. In practice, girls and boys of each age cohort in each study community were separately placed in random order by the unique personal identification number. Every *k*th girl and every *k*th boy in each community were selected so that the sample consisted of the required number of boys and girls. The varying *k* factors were determined by sample size and the total number of boys and girls in the different age cohorts in each community.

Of the 4326 subjects, 3596 (83.2%) participated in the initial survey in 1980. Since baseline, these participants were followed up on seven occasions (1983, 1986, 1989, 1992, 2001, 2007, and 2010–2011), with 2005 of the original participants (55.8%) remaining in the sample in 2011(Supplement 4). The examinations have included comprehensive data collection using questionnaires, physical measurements, and blood tests. In 2010, the participants were adults aged 33, 36, 49, 42, 45, and 48 years. Participants, or their parents, gave written informed consent and the study protocol was approved by the ethics committee of the participating universities.

### 2.2. Physical Activity and Smoking

Smoking and PA were assessed through a self-administered questionnaire five to eight times (depending on age) during the 31 years of follow-up. Questions related to PA in 1980–1989 concerned the frequency and intensity of leisure-time PA, participation in sports club training, participation in sports competitions, and habitual ways of spending leisure time starting from the age of 9 years. From the 1992 follow-up, the questions concerned the intensity of PA, frequency of vigorous PA, hours spent in vigorous PA, the average duration of a PA session, and participation in organized PA during leisure-time. The five items were recorded as inactive or very low activity (= 1) to regular or vigorous activity (= 2 or 3) and then summed to form a physical activity index (PAI; range 5–14/15) [10]. The validity of the PAI has been studied by correlating child and adult PAIs with indicators of exercise capacity [10,33] and objectively measured data [33]. Smoking information has been measured four to eight times beginning when participants were 12 years old. Participants were asked to indicate how many cigarettes of different types (factory-made, hand-rolled, pipe, cigar) they smoked per day. The total number of cigarettes per day was calculated as the sum of different types of cigarettes (range 0–30). Questions related to smoking and PA and the coding of these variables are presented in Supplementary Materials supplement 1.

### 2.3. Ethics and Consent

Study participants provided written informed consent in accordance with the Helsinki Declaration and the study protocol was reviewed and approved (88/180/2010) by the ethics committee of the participating universities (5) [34].

### 2.4. Statistical Analysis

Descriptive statistics were calculated using IBM SPSS Statistics for Windows (version 24.0) and further modeling performed using Mplus, version 7.0 [35]. Trajectories for smoking and PA over 31 years were defined using latent profile analysis (LPA). Moreover, the relationship between the trajectories was examined via multinomial logistic regression and latent transition probabilities. Participants having at least one assessment of smoking and PA over seven periods were included in the analyses, ending up with a sample of 3355 participants (47.9% men). On average, participants had 5.3 measures for smoking and PA. Multiple cohort data were reorganized in a way that each measurement point represented the participant’s age instead of measurement year. The content of the PA questionnaire was marginally different between the time intervals of 1980–1989 and 1992–2011. In order to avoid overlaps of the two scales, the PA data of the oldest cohort in 1986, the two oldest cohorts in 1989 and three oldest cohorts in 1992 were omitted (recorded as missing). PA data covered ages from 9 to 48. In addition, reorganized smoking data were averaged over three-year periods ending up with six measurement points at ages 15–18, 21–24, 27–30, 33–36, 39–42 and 45–48. Averaged smoking was treated as a censored zero-inflated normal variable in the modeling.

First, LPA was performed to identify the optimal number of latent classes for smoking and PA for both genders. The mean profiles were freely estimated and residual variances were fixed to be equal across classes. Based on previous research, one to six class models were fitted to the data. Akaike’s information criterion (AIC), Bayesian information criterion (BIC), and sample-size adjusted Bayesian information criterion (ABIC) were used to evaluate the goodness-of-fit of the LPA with a different number of classes. A model with lower values of information criteria fitted the data better than an alternative model with higher values. Furthermore, statistical tests were used to determine a sufficient number of classes: Vuong–Lo–Mendell–Rubin likelihood ratio test (VLMR), Lo–Mendell–Rubin adjusted likelihood ratio test (LMR) and parametric bootstrapped likelihood ratio test (BLRT). The estimated model was compared to the model with one class less, with a low *p* value indicating that the model with one classless was rejected in favor of the estimated model [35]. It has been shown that by identifying the correct number of classes in mixture modeling, BIC outperforms the other information criteria and BLRT the other likelihood-based tests [36]. In our study, BLRT did not differ between the different solutions for smoking or PA (supplement 2). Hence, for PA we based our judgments on BIC [36]. Among men, BIC suggested that a four-group PA solution had the best fit to the data. Among women, five groups appeared to represent the data best. The quality of the classification was evaluated using posterior probabilities for the most likely latent class membership, with an average posterior probability of 0.70 for all groups as a minimum indicator of well-separated classes [17]. Among men and women, the average posterior probabilities dropped below 0.70, when the number of groups was increased to above four groups for men and above five groups for women. For smoking, BIC and ABIC decreased for all models from one to five or six classes. However, average posterior probabilities dropped below 0.70 after the five-class model and therefore the five-class model was considered optimal.

Second, the relationship between the trajectories of smoking and PA was studied. A multinomial logistic regression model was specified between the latent class variables; that is, the latent class variable of smoking was regressed on the latent class variable of PA. The number of classes, the mean profiles, and the residual variances were fixed based on the LPAs. Latent transition probabilities, or the probability of belonging to each smoking trajectory conditional on a given PA trajectory, were obtained. The approach is similar to dual trajectory analysis.

Missing data were assumed to be missing at random (MAR). Parameters of the models were estimated by using the full information maximum likelihood (FIML) method with robust standard errors (MLR). The method produces unbiased parameter estimates under MAR assumption.

## 3. Results

The characteristics of the study participants in 2011 are presented in Table 1. Participants having at least one measurement of smoking and PA were included in the analyses (1607 males and 1748 females).

### 3.1. Physical Activity

**Figure 1:**
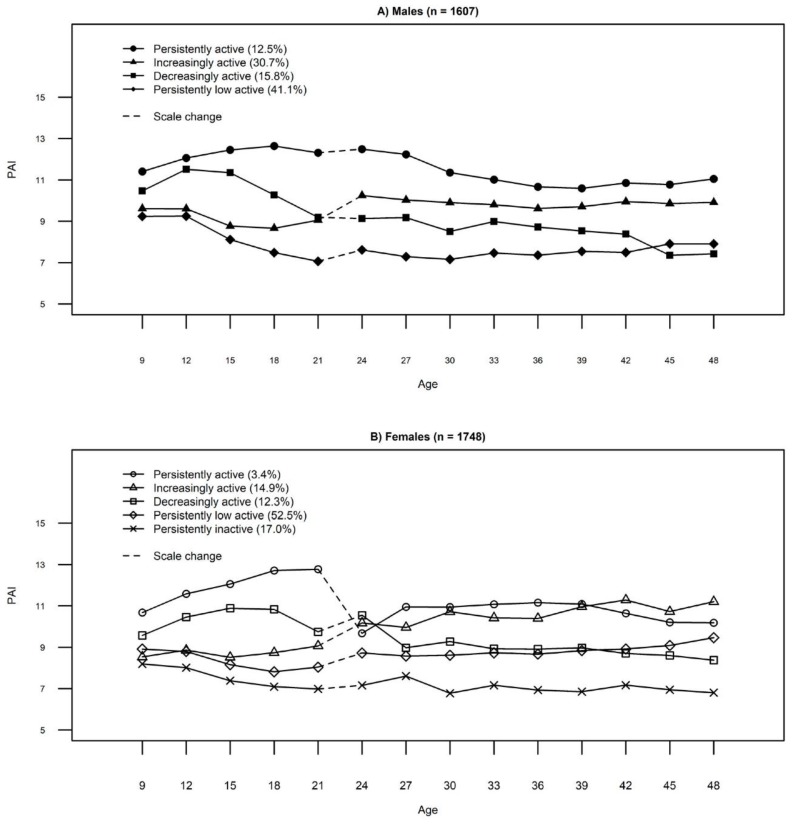
PA trajectories among (**A**) males and (**B**) females.

In LPA, four discrete PA trajectories were identified among males. They were named as follows: persistently low active (41.1%), decreasingly active (15.8%), increasingly active (30.7%), and persistently active (12.5%). Among females, five discrete PA trajectories were identified and were named as persistently inactive (17.0%), persistently low active (52.5%), decreasingly active (12.3%), increasingly active (14.9%), and persistently active (3.4%). The proportion of the study population assigned to each group and the posterior probabilities are shown in Table 2, and the mean profiles of PA subgroups are presented in Figure 1. Although some of the identified groups had low proportions of participants, they are highly discriminated with high mean posteriori probabilities, as shown in Table 2.

### 3.2. Smoking

**Figure 2:**
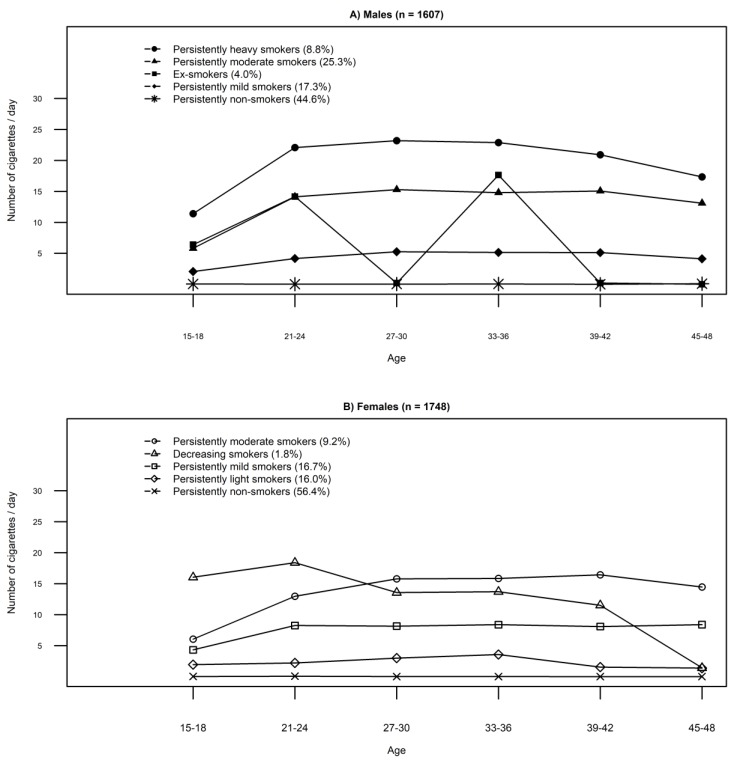
Smoking trajectories among (**A**) males and (**B**) females.

In LPA, five discrete smoking trajectories were identified for both males and females. In line with earlier research [34], the group names were based on the number of cigarettes smoked in a day: light (1–4 cigarettes daily), mild (≤10 cigarettes daily), moderate (11–20 cigarettes daily) and heavy (>20 cigarettes daily). Among both genders, three similar smoking groups were identified and named as persistently non-smokers (men, 44.6%, women, 56.4%), persistently mild smokers (17.3%, 16.7, respectively), and persistently moderate smokers (25.3%, 9.2%, respectively). Two additional groups were found for men: persistently heavy smokers (8.8%) and ex-smokers (4.0%). For females, the additional groups were decreasing smokers (1.8%) and persistently light smokers (16.0%). The proportion of the study population assigned to each trajectory group is shown in Table 2, with mean profiles of smoking subgroups shown in Figure 2. Overall, the smoking trajectories seemed to have good convergent validity (Supplement 3, Figure S1 for male and Figure S2 for female).

**Figure 3:**
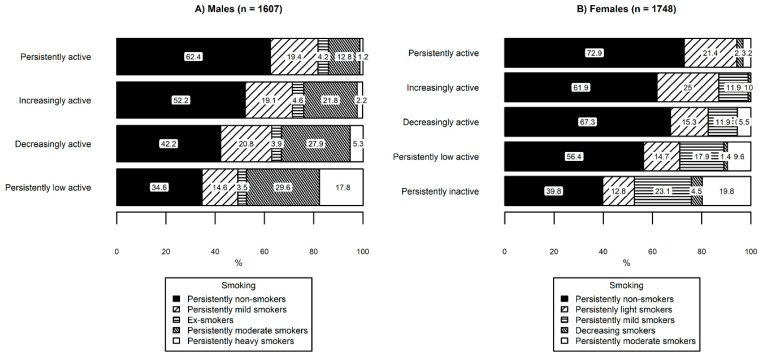
Latent transition probabilities based on the estimated model among (**A**) males and (**B**) females.

A multinomial logistic regression model between the latent class variables of PA and smoking produced similar mean profiles and class-sizes compared to independently conducted LPA models (see Table 2) for PA classes (males: 11.7%, 31.1%, 15.4%, and 41.8%; females: 3.5%, 15.0%, 12.2%, 52.5%, and 16.9%), and for smoking classes (males: 44.7%, 19.1%, 4.4%, 22.8%, and 9.1%; females: 56.3%, 16.2%, 16.5%, 1.8%, and 9.2%). For the model, the lowest PA group (a persistently low active group for males, and a persistently inactive group for females) was considered as the reference group, and the persistently non-smokers group was considered as the reference group for smoking. Structural parameters of the multinomial regression model are presented in Table 3 and latent transition probabilities for males and females are presented in Figure 3.

Among males, the persistently active group had a lower probability of belonging to the persistently heavy smokers group (*b* = −3.32, *s.e.* = 1.39, *p* = 0.017) and persistently moderate smokers group (*b* = −1.43, *s.e*. = 0.34, *p* < 0.001) than persistently low active group (Table 3). The increasingly active group had a lower probability of belonging to persistently heavy smokers group (*b* = −2.50, *s.e.* = 0.63, *p* < 0.001) and persistently moderate smokers (*b* = -0.72, *s.e.* = 0.24, *p* = 0.003) than the persistently low active group (Table 3). The decreasingly active group had a lower probability of belonging to the persistently heavy smokers (*b* = −1.41, *s.e.* = 0.58, *p* = 0.015) when compared with the low active group (Table 3).

Among females, the persistently active group had a lower probability of belonging to the persistently moderate smokers group (*b* = −2.43, *s.e.* = 1.21, *p* = 0.044) compared to the persistently inactive group and none of them belonged to the persistently mild smokers group. The increasingly active group had a lower probability of belonging to the persistently mild smokers group (*b* = −1.11, *s.e.* = 0.42, *p* = 0.007) and none of them belonged to the persistently moderate smokers group. The decreasingly active group had a lower probability of belonging to the persistently moderate smokers group (*b* = −1.80, *s.e.* = 0.53 *p* = 0.001) and persistently mild smokers group (*b* = −1.20, *s.e.* = 0.41, *p* = 0.004). In addition, none of the participants assigned to the decreasingly active group belonged to the decreasing smokers group. The persistently low active group had a lower probability of belonging to the persistently moderate smokers (*b* = −1.08, *s.e.* = 0.33, *p* = 0.001), the persistently mild smokers (*b* = 0.60, *s.e.* = 0.30, *p* = 0.043), and the decreasing smokers group (*b* = −1.54, *s.e.* = 0.73, *p* = 0.036) compared to the persistently inactive group.

## 4. Discussion

This study determined distinct trajectories of PA and smoking from childhood and adolescence to mid-adulthood for males and females in a Finnish population. While there are longitudinal studies that have determined the relationship between smoking and PA, these studies [28,30,31,32] concentrate on youth and do not provide a clear view to the lifelong smoking behavior. Although we were unable to confirm the direction of our associations or confer causality, our observational data suggest a strong relationship between smoking and PA trajectories from youth to adulthood. In particular, those who are persistently active or increasingly active have substantially reduced probabilities of being in the highest-risk smoking categories. This study shows that inactive and decreasing PA groups are the most important groups to target for intervention endeavors.

These results are in line with previous studies that suggested smoking be grouped into five distinct trajectories [23,24]. As we observed, previous studies have identified groups of non-smokers [21,22,25], stable smokers [22], heavy smokers [21,23,24], and decreasing smokers [22,24,25]. However, results in this study might have been affected by the tightening of the smoking legislation that occurred in Finland in 2006 and 2010. Since 2006, smoking has been declining among the working population and adolescents in Finland [37]. This change in legislation might have affected the smoking behavior of the study participants as well. Our identification of four PA trajectory groups in males and five in females are in line with previous studies [27,28,29]. These previous studies identified similar trajectory groups to those that we observed (e.g., persistently low activity, decreasing PA, increasing PA, persistently high activity) [27,28].

Increasing PA seemed to continue among women, while among men the trend was more or less stable. This trend in the trajectories might be explained by women being more likely to pay attention to their health habits [38]. Men are less interested in health-enhancing activities and more likely to adopt beliefs and behaviors that increase their risks [38].

The results of this study suggest that there may be an association of PA to non-smoking. Those with a continuously active, increasing PA or decreasing high PA level seemed to smoke less than those with a low or inactive level. Hence, this finding suggests that an active lifestyle from youth to adulthood could prevent smoking or that an increasing PA from youth to adulthood could reduce smoking.

When compared to the low active group, males in the decreasingly active group were at a lower probability of belonging to the persistently heavy smokers group. Even though PA is decreasing in adulthood, it may be that high PA in adolescence prevents the commencement of smoking later in life. For example, physically active youths are less likely to start smoking in adolescence [39]. The proportion of non-smokers in the group of decreasing PA versus persistently inactive in females (67.3% vs 39.8%) and versus persistently low active in males (42.2% vs 34.6%) and proportion of moderate smokers among females (5.5% vs. 19.8%) and heavy smokers among males (5.3% vs. 17.8%) supports this explanation that people in the declining PA group have acquired their non-smoking lifestyle or habit of smoking less earlier in their life. Nevertheless, among the decreasingly active group, there were no decreasing smokers among females.

When looking at the PA profiles more closely, it can be seen that they remain stable after the age of 24 years. Rapid or steep increases or decreases were not observed. However, the birth of a child, changes in working life or in marital status may change PA behavior [40]. On the other hand, after 18 years of age, there is a decrease, especially among men. This may be related to car access, since the age for a drivers license in Finland is 18 years. In previous studies holding a driver’s license had a negative effect on PA [41]

Among females, smoking seems to be a more dynamic habit including more changes during the lifespan, while among males it seems to be more stable. Between the ages of 18 to 21 years, there is mainly an increase in smoking, but after this period, smoking behavior seems to stabilize. Earlier research has also found the stabilization of smoking after 21 years [11]. This would suggest a sensitive or critical period in the life-course when prevention might be most effective. Earlier literature states that smoking prevalence increases strongly between the ages of 15 and 21 [11] or even before [28]. Coinciding with this same age range, most Finnish men participate in compulsory military service. Military service, therefore, could be a potential period for smoking prevention because most of the age cohort participate in the service. However, current available evidence suggests military service to be a more encouraging environment for smoking [42].

There is also clear evidence of the integrational transmission of smoking. That is why it is essential to influence smoking across the lifespan [43]. Linking smoking cessation with other good health habits is important not only for adults but also for future generations.

Traditionally, public health promotion tends to target a single health behavior. Yet unhealthy behaviors tend to cluster, so public health promotion should target several health behaviors at the same time [14]. In Finland, several different agencies currently target PA, smoking, or drinking. However, it might be more efficient and effective if these agencies work together to promote a broader, more holistic view of healthy behaviors. In addition, understanding different distinct trajectories of smoking and PA could also provide possibilities to promote specific intervention for certain PA or smoking profiles. Further research is needed to understand what kind of intervention would be most beneficial for different PA or smoking profiles.

Further research should aim to identify the overall effect of PA behavior on a cluster of health habits. The longitudinal study with seven follow-up points is the strength of this study. In addition, the six different cohorts provided more support for the results. However, smoking and PA have been reported with a self-reported questionnaire and so there may be some under- or over-reporting. However, the questionnaire used for PA in this study correlates well with objectively measured PA [33].

## 5. Conclusions

The trajectories of PA and smoking from youth to adulthood vary for males and females, with trajectories associated with PA shown to be associated with trajectories of smoking behavior over the same period. Females and males who belonged to the increasing or decreasing PA trajectories had a lower probability of being smokers compared with those in the physically inactive or low active groups. Despite showing a decrease in PA levels from youth to adulthood, those in the decreasing PA group seemed to exhibit a lasting effect of higher youth PA on smoking behavior in adulthood. These findings offer new insights into the relationship between smoking and PA across late adolescence to mid-adulthood.

## Figures and Tables

**Table 1:** Characteristics of the study sample, year 2011.

	Males (*N* = 1607, 47.9%)	*n*	Females (*N* = 1748, 52.1%)	*n*
Age (years), mean (SD)	41.7 (4.9)		41.7 (4.9)	
Height (cm), mean (SD)	179.8 (6.6)	923	166.1 (6.0)	1115
Weight (kg), mean (SD)	87.5 (16.0)	923	72.0 (15.4)	1117
BMI, mean (SD)	27.0 (4.4)	922	26.1 (5.5)	1114
Education (%)		878		1096
≤12 years	34.3		18.4	
>12 years	65.7		81.6	
SES (%)		801		981
Manual	33.3		8.8	
Non-manual, low	20.8		53.1	
Non-manual, high	45.8		38.1	
Smoking status 2011 (%)		882		1105
Non-smoker	78.6		84.3	
Smoker	21.4		15.7	
Physical activity 2011 (%)		877		1095
Once a month or seldom	24.2		17.4	
Once a week	22.2		23.7	
2–3 times a week	38.3		38.4	
4–6 times a week	13.2		16.5	
Every day	2.1		4.0	

**Table 2:** Trajectory group distributions and posterior probabilities for males and females.

	Distribution %	Posterior Probability (M)
PA trajectory group		
Males		
Group 1: Persistently active	12.5	0.87
Group 2: Increasingly active	30.7	0.78
Group 3: Decreasingly active	15.8	0.74
Group 4: Persistently low active	41.1	0.83
Females		
Group 1: Persistently active	3.4	0.85
Group 2: Increasingly active	14.9	0.75
Group 3: Decreasingly active	12.3	0.78
Group 4: Persistently low active	52.5	0.78
Group 5: Persistently inactive	17.0	0.79
Smoking trajectory group		
Males		
Group 1: Persistently non-smokers	44.6	0.91
Group 2: Persistently mild smokers	17.3	0.83
Group 3: Ex-smokers	4.0	0.72
Group 4: Persistently moderate smokers	25.3	0.76
Group 5: Persistently heavy smokers	8.8	0.82
Females		
Group 1: Persistently non-smokers	56.4	0.94
Group 2: Persistently light smokers	16.0	0.78
Group 3: Persistently mild smokers	16.7	0.76
Group 4: Decreasing smokers	1.8	0.91
Group 5: Persistently moderate smokers	9.2	0.81

**Table 3:** Structural parameters of the multinomial logistic regression model of PA and smoking among males (*n* = 1607) and females (*n* = 1748).

	**Smoking among Males (Non-Smokers as Reference Group)**											
	**Persistently Heavy Smokers**			**Persistently Moderate Smokers**			**Ex-Smokers**			**Persistently Mild Smokers**		
	***b***	***s.e.***	***p***	***b***	***s.e.***	***p***	***b***	***s.e.***	***p***	***b***	***s.e.***	***p***
Persistently low active (as reference group)												
Persistently active	−3.32	1.39	0.017 *	−1.43	0.34	<0.001 ***	−0.39	0.69	0.571	−0.30	0.31	0.680
Increasingly active	−2.50	0.63	<0.001 ***	−0.72	0.24	0.003 **	−0.12	0.60	0.845	−0.14	0.27	0.593
Decreasingly active	−1.41	0.58	0.015 *	−0.26	0.28	0.364	−0.08	0.86	0.923	0.16	0.33	0.626
	**Smoking among Females (Rare or Non-Smokers as Reference Group)**											
	**Persistently Moderate Smokers**			**Persistently Mild Smokers**			**Decreasing Smokers**			**Persistently Light Smokers**		
	***b***	***s.e.***	***p***	***b***	***s.e.***	***p***	***b***	***s.e.***	***p***	***b***	***s.e.***	***p***
Persistently inactive (as reference group)												
Persistently active	−2.43	1.21	0.044 *	- ^a^	-	-	−1.20	1.28	0.734	0.09	0.52	0.865
Increasingly active	- ^a^	-	-	−1.11	0.42	0.007**	−1.77	1.04	0.090	0.23	0.39	0.555
Decreasingly active	−1.80	0.53	0.00 1**	−1.20	0.41	0.004**	-	-	-	−0.35	0.44	0.438
Persistently low active	−1.08	0.33	0.001 **	−0.60	0.30	0.043*	−1.54	0.73	0.036*	−0.21	0.38	0.578

Note. ^a^ There was an empty cell in joint distribution of latent class variables. The parameter estimates could not be determined. *b*, regression coefficient; *s.e.*, standard error. Significant difference between groups; * *p <* 0.005, ** *p* < 0.01, *** *p* < 0.001.

## Supplementary Materials

The following are available online at https://www.mdpi.com/1660-4601/16/6/974/s1, Supplement Digital Content 1: Young Finns study questionnaire. Questions related to physical activity and smoking, and coding between the years 1980–2011. Supplement Digital Content 2: The model fit and class sizes from the latent profile analyses of physical activity (PA) and smoking among males (*n* = 1607) and among females (*n* = 1748). Supplement Digital Content 3: Convergent validity of smoking trajectories. Supplement 4: Flow chart of the study
