# Supplementary material for: Smoking and Physical Activity Trajectories from Childhood to Midlife
Source: Int J Environ Res Public Health. 2019 Mar 18;16(6):974. doi: 10.3390/ijerph16060974 (PMC6466084; doi:10.3390/ijerph16060974)

International Journal of Environmental Research and Public Health

Supplement 1

Table 1. Young Finns study questionnaire. Questions related to physical activity and smoking, and coding between the years 1980–2011.

Years 1980–1989

	Coding
How often do you engage in leisure-time physical activity at least half an hour per time?	
Not at all	1
Less than once a month	1
Once a month	1
2–3 times a month	1
Once a week	2
2–6 times a week	2
Every day	3
How much are you breath-taking and sweating when you engage in physical activity and sport?	
Not at all	1
Moderately	2
A lot of	3
How many times a week do you usually engage in training sessions organized by a sport club?	
Not at all	1
Occasionally	1
Less than once a month	1
Once a month or more	2
Once a week	2
Several hours and times a week	3
Do you participate in sports competitions?	
Not at all	1
Sports club level	2
Regional level	2
National level	2
What do you usually do in your leisure time?	
I am usually indoors and read or do other sedentary activities	1
I spend my time indoors and outdoors	1
I usually walk or spend my time with friends	2
I am usually outdoors and exercise quite a lot	3
Count	5-14

Years 1992-2011

	Coding
How often do you engage in rigorous physical activity?	
Not at all	1
Once a month or more	1
Once a week	1

2–3 times a week<br>4–6 times a week<br>Every day	2<br>2<br>3
How much breathlessness and sweating do you experience when you engage in physical activity and sport<br>Not at all<br>Moderate amount<br>A lot	 <br>1<br>2<br>3
How many hours per week do you engage in rigorous physical activity?<br>Not at all<br>30 minutes<br>1 hour<br>2–3 hours<br>4–6 hours<br>7 hours or more	 1<br>1<br>1<br>2<br>2<br>3
How much time do you usually spend in a physical activity session?<br>Less than 20 minutes<br>20–40 minutes<br>40–60 minutes<br>>60 minutes	 1<br>2<br>2<br>3
Do you participate in organized physical activity?<br>Not at all<br>Occasionally<br>Regularly, about once per week<br>Several hours and times per week	 1<br>1<br>2<br>3
Count	5-15

Years 1980–2011

Daily smoking (factory-made)<br>Daily smoking (self-made)<br>Daily smoking (pipeful)<br>Daily smoking (cigar)	Count<br>Count<br>Count<br>Count
	Sum

Table S2. The model fit and class sizes from the latent profile analyses of physical activity (PA) and smoking among males (n = 1607) and among females (n = 1748).

Number of classes	AIC	BIC	ABIC	VLMR	LMR	BLRT	Class sizes [a]	AvePP
PA								
Men								
1	31213	31364	31275	-	-	-	-	-
2	29762	29994	29857	<0.001	<0.001	<0.001	73.7%, 26.3%	0.95, 0.91
3	29443	29756	29571	0.009	0.010	<0.001	43.4%, 39.8%, 16.8%	0.84, 0.79, 0.89
**4**	**29238**	**29631**	**29399**	**<0.001**	**<0.001**	**<0.001**	**41.1%, 30.7%, 15.8%, 12.5%**	**0.83, 0.78, 0.74, 0.87**
5	29185	29659	29380	0.300	0.305	<0.001	32.8%, 23.5%, 16.5%, 15.6%, 11.5%	0.67, 0.77, 0.75, 0.74, 0.87
6	29128	29682	29355	0.476	0.479	<0.001	31.5%, 17.1%, 16.5%, 15.4%, 10.2%, 9.4%	0.66, 0.73, 0.68, 0.72, 0.83, 0.76
Women								
1	34265	34418	34329	-	-	-	-	-
2	33147	33382	33245	<0.001	<0.001	<0.001	84.1%, 15.9%	0.97, 0.89
3	32780	33097	32913	<0.001	<0.001	<0.001	57.4%, 30.8%, 11.8%	0.83, 0.81, 0.90
4	32660	33059	32827	0.689	0.690	<0.001	49.5%, 33.8%, 12.5%, 4.2%	0.80, 0.80, 0.77, 0.83
**5**	**32531**	**33012**	**32732**	**0.202**	**0.203**	**<0.001**	**52.5%, 17.0%, 14.9%, 12.3%, 3.4%**	**0.78, 0.79, 0.75, 0.78, 0.85**
6	32477	33040	32713	0.746	0.747	<0.001	39.9%, 17.3%, 16.6%, 12.4%, 10.3%, 3.4%	0.68, 0.76, 0.68, 0.72, 0.75, 85.4
Smoking								
Men								
1	18092	18188	18131	-	-	-	-	-
2	16634	16768	16689	<0.001	<0.001	<0.001	50.3%, 49.7%	0.93, 0.97
3	16293	16465	16364	<0.001	<0.001	<0.001	44.5%, 33.2%, 22.3%	0.91, 0.89, 0.82
4	16215	16425	16301	0.063	0.067	<0.001	44.4%, 28.3%, 18.4%, 8.9%	0.94, 0.78, 0.79, 0.86
**5**	**16191**	**16438**	**16292**	**0.048**	**0.051**	**<0.001**	**44.6%, 25.3%, 17.3%, 8.8%, 4.0%**	**0.91, 0.76, 0.83, 0.82, 0.72**
6	16137	16422	16254	0.092	0.095	<0.001	44.8%, 15.8%, 13.7%, 10.0%, 9.9%, 5.7%	0.91, 0.64, 0.64, 0.67, 0.70, 0.79
Women								

1	17208	17306	17249	-	-	-		-
2	15332	15469	15389	<0.001	<0.001	<0.001	62.5%, 37.5%	0.96, 0.96
3	15043	15218	15116	0.040	0.050	<0.001	58.8%, 25.3%, 15.9%	0.95, 0.84, 0.87
4	14950	15163	15039	0.285	0.289	<0.001	56.2%, 18.4%, 16.7%, 8.7%	0.94, 0.78, 0.79, 0.86
**5**	**14903**	**15154**	**15008**	**0.012**	**0.013**	**<0.001**	**56.4%, 16.7%, 16.0%, 9.2%, 1.8%**	**0.94, 0.76, 0.78, 0.81, 0.91**
6	14897	15187	15019	0.111	0.115	0.050	55.9%, 15.8%, 12.9%, 9.1%, 4.4%, 1.8%	0.94, 0.75, 0.74, 0.61, 0.92

[a] Final class counts and proportions for the latent class patterns based on estimated posterior probabilities.
AIC, Akaike's information criterion; BIC, Bayesian information criterion; ABIC, sample-size adjusted Bayesian information criterion; VLMR, Vuong-Lo-Mendell-Rubin likelihood ratio test; LMR, Lo-Mendell-Rubin adjusted LRT test; BLRT, Parametric bootstrapped likelihood ratio test.
AvePP, average posterior probabilities for most likely latent class membership

Supplement S3. Convergent validity of smoking trajectories.

Convergent validity of the smoking trajectories was visually inspected. For that purpose, participants were classified based on their most likely latent class membership. Besides of the number of daily smoked cigarettes, participants were asked to report their current smoking status. According to the self-reported smoking status, participants were classified to 1 = non-smokers, 2 = occasional smokers and 3 = regular smokers (smokes at least once a day) at each age point. The proportion of the non-smokers and the regular smokers over ages in males are presented in Figure S1 and in females in Figure S2. Among males, the proportion of the non-smokers was constantly highest within the persistently non-smokers group (contsantly over 80%). Similarly, the proportion of the regular smokers was constantly highest within the persistently heavy smokers group. Within the ex-smokers group the proportion of the regular smokers was highest at ages of 18 and 21 (over 70%), and proportion of the non-smokers was over 70% from the age of 27 years.

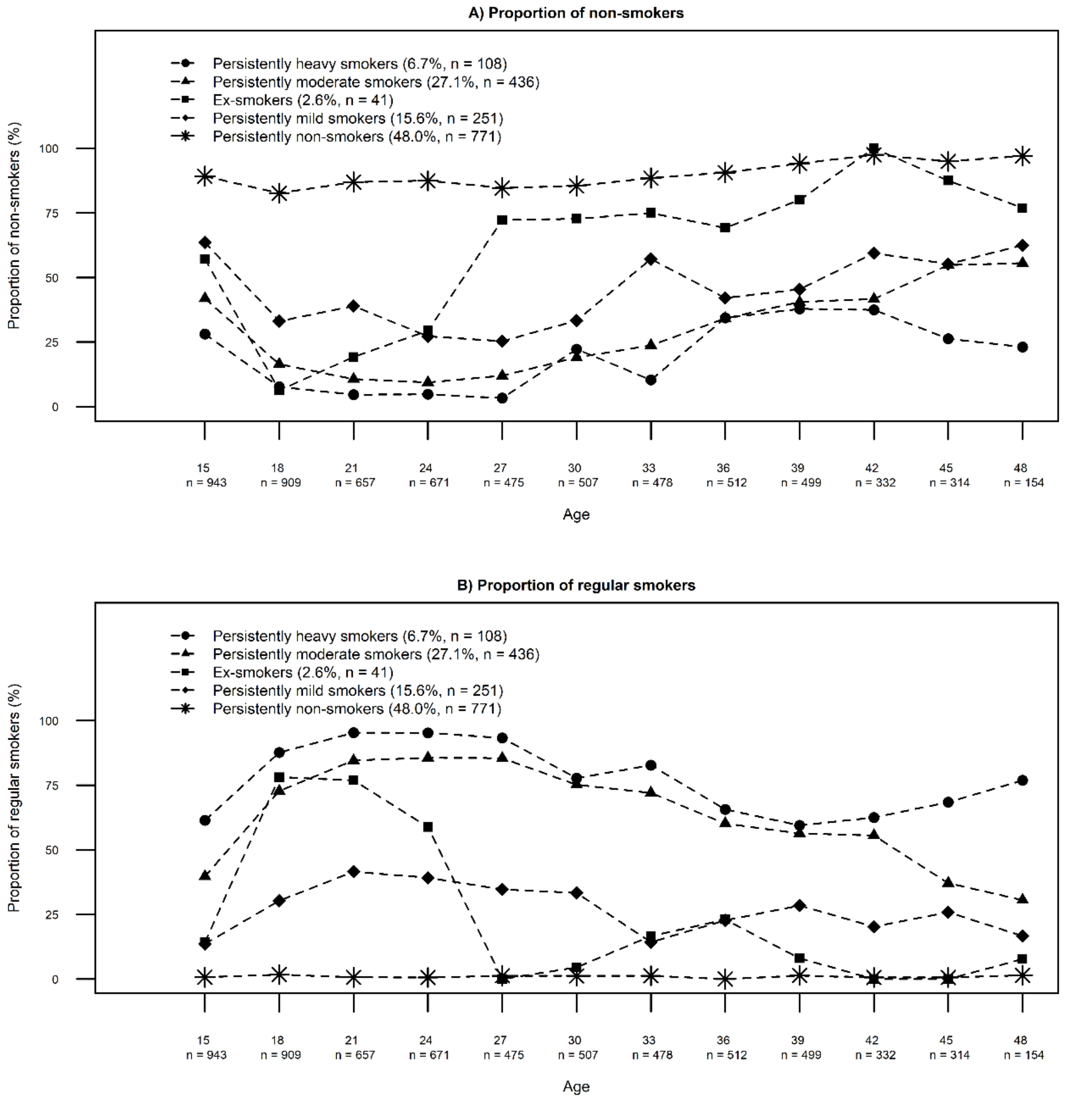

Figure S1. The proportion of the non-smokers (**A**) and the regular smokers (**B**) over ages among males.

Among females, the proportion of non-smokers was constantly highest within the persistently non-smokers group (constantly over 80%). The proportion of the regular smokers was constantly

higher within the persistently moderate smokers group compared to the persistently mild, light and non-smokers group. The proportion of regular smokers within the marginal group of decreasing smokers seemed to fluctuate.

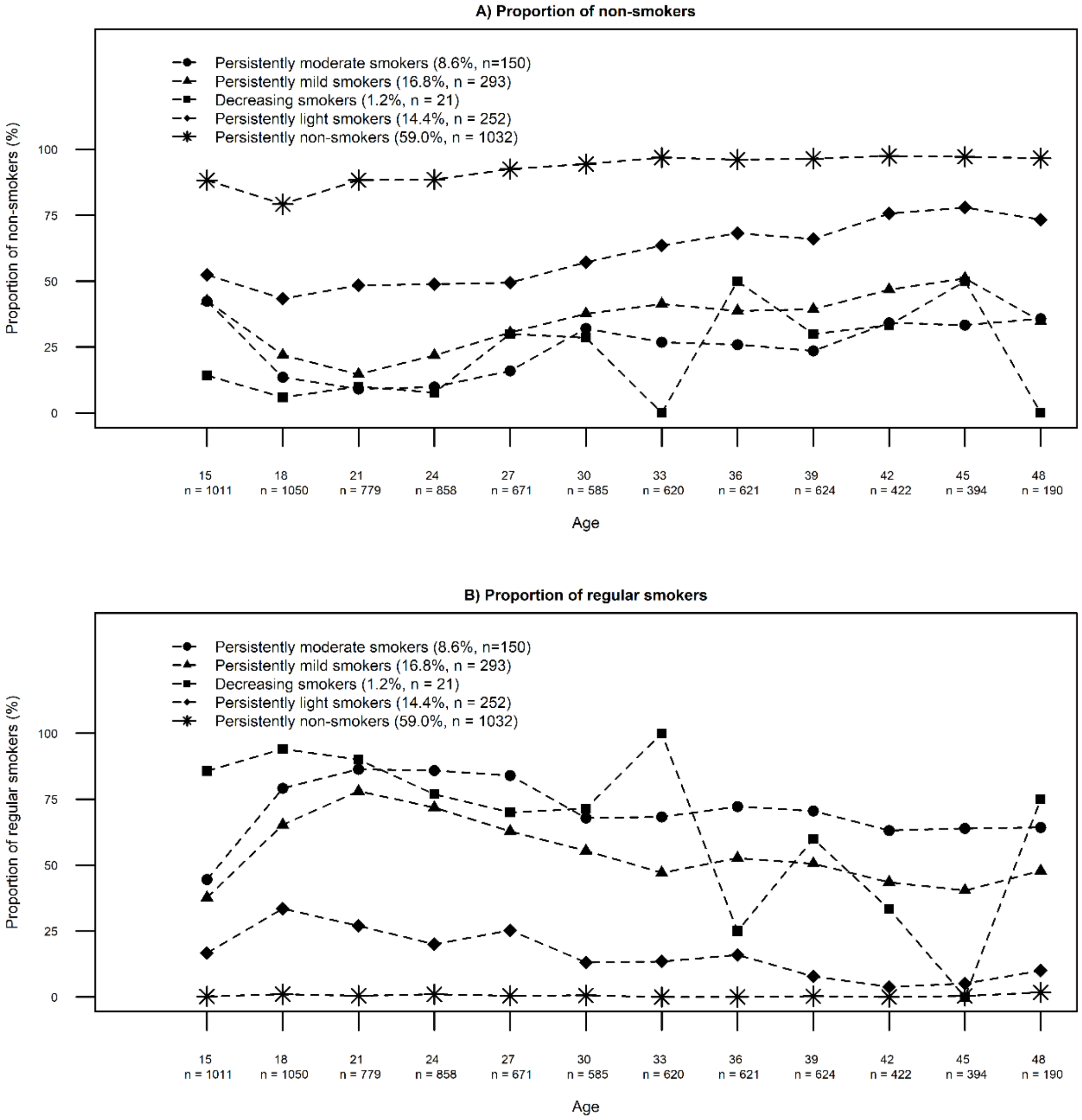

Figure S2. The proportion of the non-smokers (**A**) and the regular smokers (**B**) over ages among females.

Supplement 4. Flow chart of the study.

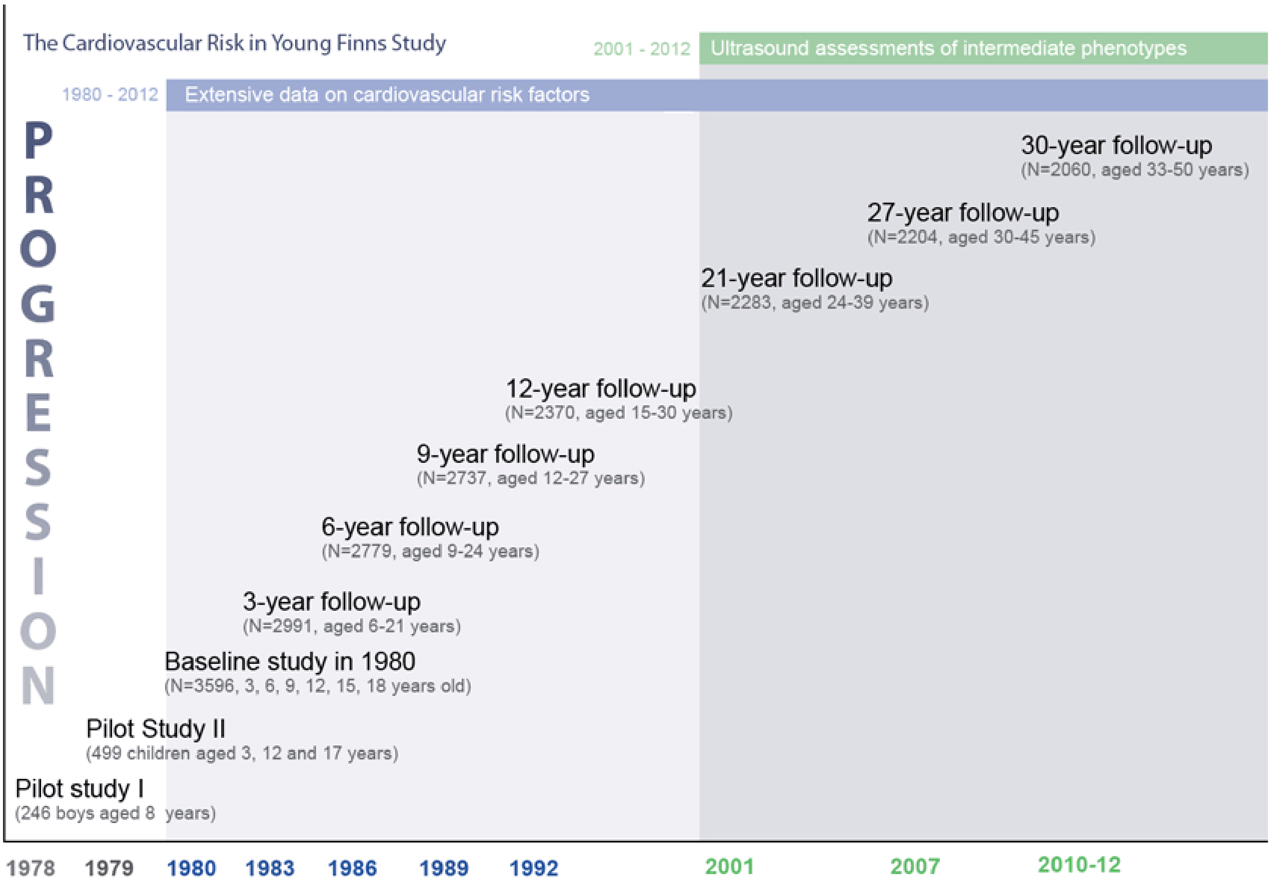

Supplement: Supplementary file 1 [file ijerph-16-00974-s001.pdf]
